# Metastatic insulinoma: exploration from clinicopathological signatures and genetic characteristics

**DOI:** 10.3389/fonc.2023.1109330

**Published:** 2023-05-12

**Authors:** Jingcheng Zhang, Rui Jiang, Xiafei Hong, Huanwen Wu, Xianlin Han, Wenming Wu

**Affiliations:** ^1^ Department of General Surgery, Peking Union Medical College Hospital, Chinese Academy of Medical Science & Peking Union Medical College, Beijing, China; ^2^ Department of Pathology, Peking Union Medical College Hospital, Chinese Academy of Medical Science & Peking Union Medical College, Beijing, China; ^3^ State Key Laboratory of Complex Severe and Rare Diseases, Peking Union Medical College Hospital, Chinese Academy of Medical Science & Peking Union Medical College, Beijing, China

**Keywords:** genomic sequencing, insulinoma, malignant, metastasis, pancreatic neuroendocrine tumour

## Abstract

**Background:**

Insulinoma is a rare type of pancreatic neuroendocrine tumor with low incidence and low-malignant features. While very few insulinomas present with malignant behaviours, such as lymph node and liver metastasis, only a few studies have focused on this field owing to the limitation of samples. Existing evidence suggests that metastatic insulinoma largely derive from non-functional pancreatic neuroendocrine tumor. However, we found a portion of metastatic insulinomas may derive from non-metastatic insulinomas and explored their clinicopathological signatures and genetic characteristics.

**Methods:**

Four metastatic insulinoma patients with synchronous liver metastasis or lymph node metastasis at the Peking Union Medical College Hospital between October 2016 and December 2018 were enrolled, and whole exon and genome sequencing were performed on fresh frozen tissues and peripheral blood samples. Clinicopathological information and genomic sequencing results were collected and matched to explore the characteristics of the metastatic insulinomas.

**Results:**

These four metastatic insulinoma patients underwent surgery or interventional therapy, and their blood glucose levels immediately increased and maintained within standard range after treatment. For these four patients, the proinsulin/insulin molar ratio <1 and primary tumors were all present as PDX1+, ARX-, and insulin+, which were similar to non-metastatic insulinomas. However, the liver metastasis showed PDX1+ and ARX+, insulin+. Meanwhile, genomic sequencing data showed no recurrently mutations and typical CNV patterns. However, one patient harboured the *YY1* T372R mutation, a recurrently mutated gene in non-metastatic insulinomas.

**Conclusions:**

A portion of metastatic insulinomas were largely derived from non-metastatic insulinomas in hormone secretion and ARX/PDX1 expression patterns. Meanwhile, the accumulation of ARX expression may be involved in the progression of metastatic insulinomas.

## Introduction

Insulinoma is a rare type of pancreatic neuroendocrine tumors (PanNETs), with a reported incidence of 1–4 cases per million persons per year (1–4). Insulinoma patients suffer from hypoglycaemia and neuropsychiatric symptoms, including sweating, confusion, amaurosis, and fainting (5, 6). Surgery is a common treatment for relieving symptoms (7), but partial pancreatectomy or enucleation of insulinoma may fail to relieve hypoglycaemia symptoms (8). The 5-year survival rate of insulinomas is approximately 97% because most insulinomas present with relatively benign biological behaviour (9). Nevertheless, very few insulinomas present with malignant behaviours, such as lymph node and liver metastasis. These patients could rely on second-line treatment (such as radiofrequency ablation and octreotide therapy for liver metastasis) to control tumor progression and hypoglycaemia symptoms. Several studies have discussed the clinical treatment of metastatic insulinomas. Enucleation is not recommended because of the potential risk of tumor metastasis; thus, thorough dissection of the primary focus and resectable liver metastasis is a better recommendation (10). In addition, some studies have suggested that tumor size may be related to malignancy, indicating that the larger tumor may have higher potential to metastasize (11).

The molecular mechanisms of metastatic insulinomas remain unclear because of their low incidence. Previous studies have suggested that metastatic insulinoma is mostly derived from NF-PanNETs, with ARX being the specific transcription factor (12). However, non-metastatic insulinomas tend to express PDX1, a specific transcription factor of islet β cells. Interestingly, a large proportion of patients have no history of non-metastatic insulinomas, while a portion of metastatic insulinoma patients have a history of NF-PanNET (8, 13). Existing evidence suggests that metastatic insulinoma may largely derive from NF-PanNETs, but this needs further validation.

PanNETs is heterogenous, and the primary tumor and metastatic foci may embrace different characteristics, such as genetic and hormone expression pattern (14). Therefore, genetic sequencing and immunohistochemical staining should be performed for both the primary tumor and metastasis. In this study, four patients with metastatic insulinoma at the Peking Union Medical College Hospital (PUMCH) were enrolled. The purpose of this study was to investigate the clinicopathological features, cell origin, and genetic characteristics of metastatic insulinomas.

## Method

### Patient inclusion and retrieval of clinical information

Four metastatic insulinoma patients with synchronous lymph node or liver metastasis at the Puking Union Medical College Hospital (PUMCH) between October 2016 and December 2018 were enrolled. The inclusion criteria for metastatic insulinoma were as follows: 1) clinical diagnoses of insulinomas according to published criteria (15), 2) pathological diagnoses of PanNETs, and 3) PanNET metastasis to lymph nodes or distant organs, such as liver. We recorded detailed clinicopathological information, including 1) general information, such as sex, age at disease onset, and diagnosis; 2) qualitative diagnostic information, such as blood glucose and insulin levels; 3) imaging workups, including computed tomography (CT) and magnetic resonance imaging (MRI); 4) pathological information, such as tumor size, tumor staging, and immunohistochemical staining; and 5) treatments, including surgery, chemotherapy, arterial embolization, and microwave ablation. Follow-up visits were performed by phone calls, during which the patients stated their conditions.

### Haematoxylin-eosin and immunohistochemical staining

Tissue samples were fixed in 4% paraformaldehyde (Sigma-Aldrich, Cat# 158127) overnight, dehydrated through a serial alcohol gradient, and embedded in paraffin. Before immunostaining, tissue sections were dewaxed in xylene, rehydrated using decreasing concentrations (100%, 95%, and 75%) of ethanol, and washed in phosphate buffered saline (PBS). The sections were then stained with haematoxylin and eosin (H&E).

Immunohistochemistry (IHC) for ARX (Novus, AF7068-SP), PDX1 (Abcam, ab134150), and insulin (Cell Signalling Technology, 4590S) was performed on insulinoma tissue sections. First, the antigens were unmasked by microwaving in 10 mmol/L citrate buffer, pH 9.0, then endogenous peroxide activity was blocked with 3% peroxidase, and the slides were incubated with 10% fetal bovine serum (FBS) to prevent nonspecific binding. The slides were then incubated with primary antibodies at an optimal dilution (ARX 1:20, PDX1 1:400, and insulin 1:100) and appropriate secondary antibodies. The 3,3’-diaminobenzidine (DAB) was used to detect antibody staining. After H&E and immunohistochemical staining, the tissue sections were dehydrated using increasing concentrations (95% and 100%) of ethanol and xylene.

For ARX and PDX1, intermediate/strong nuclear staining > 10% or weak nuclear staining >50% of cells was considered positive staining. For insulin, positive staining was defined as cytoplasmic staining >10% of cells. Meanwhile, pancreatic normal islets were used as both positive and negative controls (12, 16).

### Genomic sequencing and analysis

Whole genome sequencing (WGS) and whole exon sequencing (WES) libraries were constructed for fresh frozen tissues and peripheral blood samples for patients MI1 and MI2. The constructed libraries were sequenced based on BGISEQ-500 sequencing platforms, the following process including alignment to the human reference 19 genome (hg19), sorting, and duplicate marking of high-quality sequencing reads were analysed through DRAGEN software as previously described (17). The genomic sequencing data of two patients (MI3 and MI4) were sequenced and analysed in our previous study, and we downloaded the data from the China National GeneBank Nucleotide Sequence Archive with accession number CNP0000383 (https://db.cngb.org/cnsa/) (18). Then, the downloaded files and PUMCH datasets were combined for subsequent analyses.

Somatic mutations were identified for single nucleotide variants (SNVs) by MuTect (http://www.broadinstitute.org/cancer/cga/mutect) (19) and short insertions and deletions by Platypus (http://www.well.ox.ac.uk/platypus) (20), and annotated with Oncotator (http://www.broadinstitute.org/oncotator/) (21). The integer copy number variations (CNVs) were estimated using FACETS (22), and CNV status was determined by the threshold of >2 copies for amplification and <2 copies for deletion.

## Results

### Clinical presentation, diagnosis and treatment procedures

**Figure 1:**
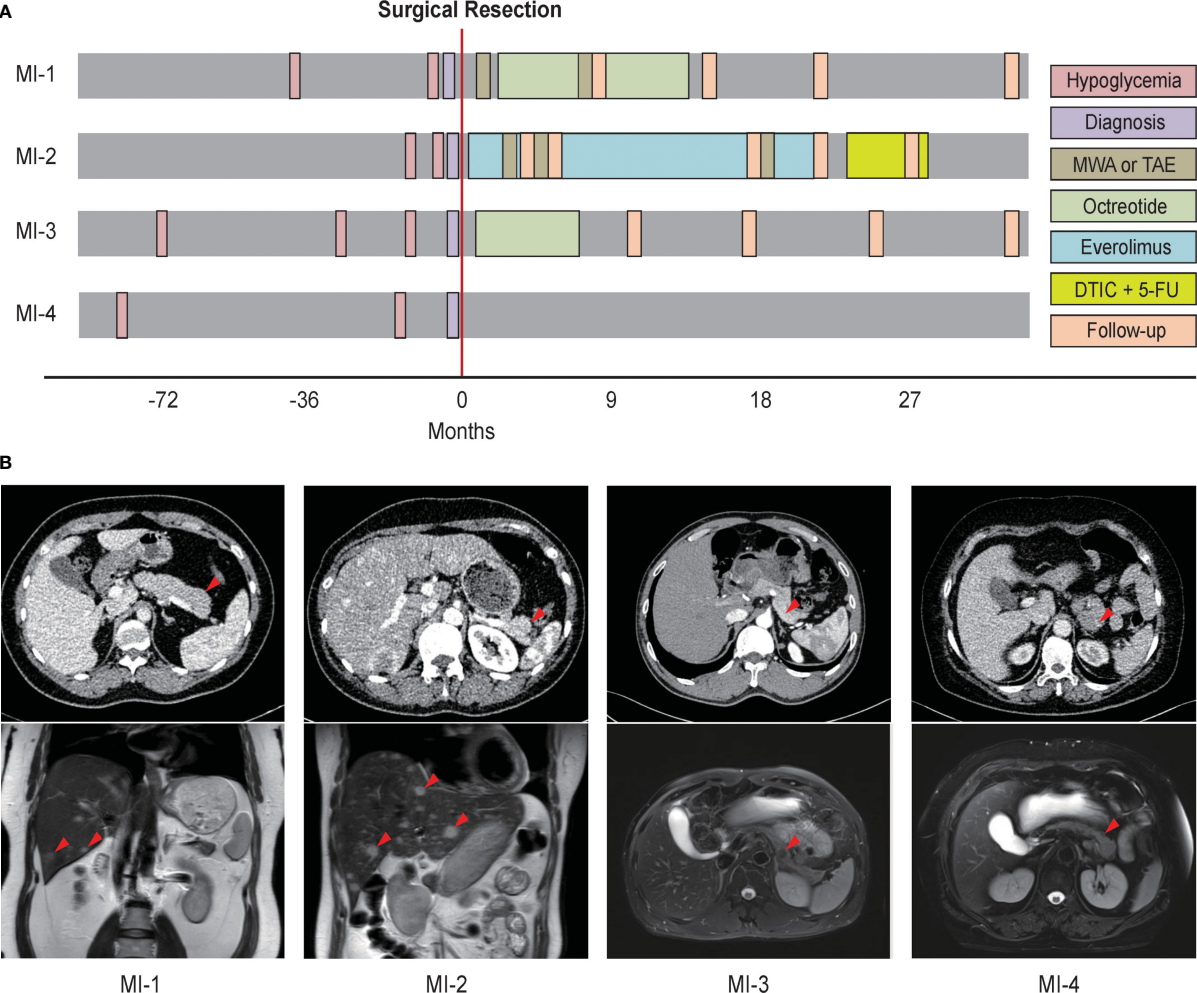
The imaging workups, diagnosis, and treatment of four patients with metastatic insulinoma. **(A)** Clinical evolution of four metastatic insulinoma patients. **(B)** The imaging report for primary tumors, lymph node metastasis, and liver metastasis of four metastatic insulinoma patients. MWA, microwave ablation. TAE, transcatheter arterial embolization. DTIC, dacarbazine. 5-FU, fluorouracil.

The four metastatic insulinoma patients all suffered from unconsciousness, and were diagnosed with insulinoma by imaging workups and typical hypoglycemia symptoms. Two patients with synchronous liver metastasis were detected by dynamic contrast-enhanced MRI, while other two patients with lymph node metastasis were verified by pathological reports (**Figure 1B**). All patients received surgery and chemotherapy (such as octreotide and everolimus) after surgery, while one patient lost follow-up. Meanwhile, patients with liver metastasis also received microwave ablation and arterial embolization for metastatic foci. The baseline characteristics and clinical evolution of each patient are summarised (**Figure 1A** and **Table 1**).

**Table 1:** Baseline characteristics of malignant insulinoma patients.

	MI-1	MI-2	MI-3	MI-4
**Age (year)**	44	49	67	50
**Gender**	Female	Female	Female	Male
**Tumor location**	Body	Tail	Tail	Tail
**Inherited syndrome**	Sporadic	Sporadic	Sporadic	Sporadic
**Baseline blood glucose (mmol/L)**	1.5	2.4	2.7	2.4
**Baseline insulin (pmol/L)**	405.54	273.18	154.66	130.28
**Baseline proinsulin (pmol/L)**	347.60	195.85	160.49	22.36
**Tumor size (cm)**	0.7	3.0	4.5 (Multiple)	1.7
**Grade**	G2	G2	G2	G1
**Ki-67 index**	3%	15%	5%	1%
**Mitotic index**	1 per 10 HPFs	8 per 10 HPFs	2 per 10 HPFs	<2 per 10 HPFs
**Metastatic Site**	Liver	Lymph node, Liver	Lymph node	Lymph node

HPFs, high-power fields.

**Figure 2:**
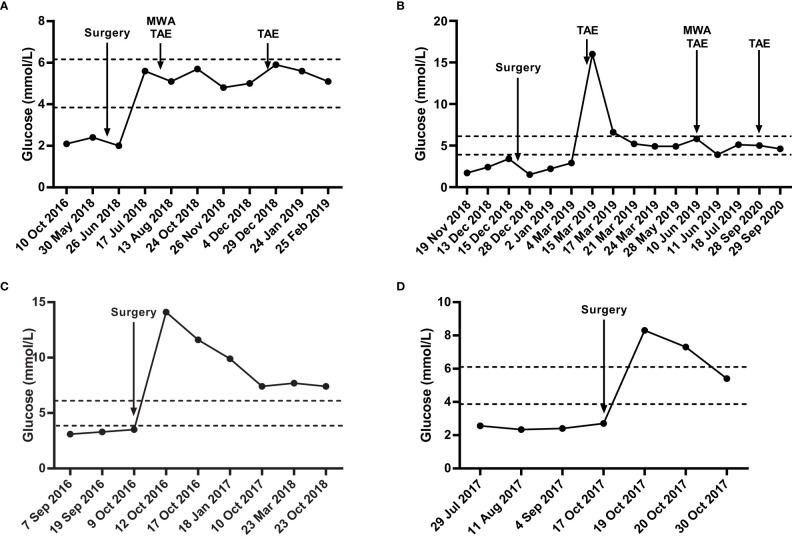
The blood glucose levels of four metastatic insulinoma patients. Blood glucose levels of MI1 **(A)**, MI2 **(B)**, MI3 **(C)**, MI4 **(D)** during surgical and chemotherapy treatment. The area among the dotted lines indicates the standard range of blood glucose level (3.9 mmol/L – 6.1 mmol/L). MWA, microwave ablation. TAE, transcatheter arterial embolization.

After surgical resection of pancreatic lesion, blood glucose levels of patients MI1, MI3, MI4 immediately increased and basically maintained within standard range. However, the blood glucose level of patient MI2 with synchronous liver metastasis was still lower than standard range, which immediately increased and maintained within standard range after treatment of liver metastasis, implying the insulin secretion capability of liver metastasis (**Figure 2**). Besides, all patients have been clinically excluded from the diagnosis of MEN-1 syndrome.

### Clinicopathological and genetic characteristics of metastatic insulinoma

The derivation of metastatic insulinomas is still unclear from existing studies. Previous studies have verified that metastatic insulinoma assembles NF-PanNETs from four aspects: natural history, circulating biochemical profile, gene expression, and treatment strategy (13). Therefore, we explored the clinicopathological characteristics mentioned above of the four metastatic insulinomas. In addition, we performed genomic sequencing to uncover the genetic signatures of metastatic insulinomas.

### Clinical characteristics

All four patients had no history of NF-PanNETs but a long-term duration of insulinomas before the final diagnosis, which could be concluded from the typical hypoglycaemia symptoms and multiple imaging examinations.

Previous studies have reported that patients with metastatic insulinomas have elevated proinsulin levels (>1000 pmol/L) and a high proinsulin/insulin molar ratio (>3), while non-metastatic insulinomas always present proinsulin levels <200 pmol/L and proinsulin/insulin molar ratio of nearly 1 (8, 23). In our study, the proinsulin and insulin levels were 347.60 pmol/L and 405.54 pmol/L for MI1, 195.85 pmol/L and 273.18 pmol/L for MI2, 160.49 pmol/L and 154.66 pmol/L for MI3, and 22.36 pmol/L and 130.28 pmol/L for MI4. The proinsulin/insulin molar ratios were 0.96, 0.89, 1.04, and 0.17, respectively.

### IHC staining results and genomic analysis

**Figure 3:**
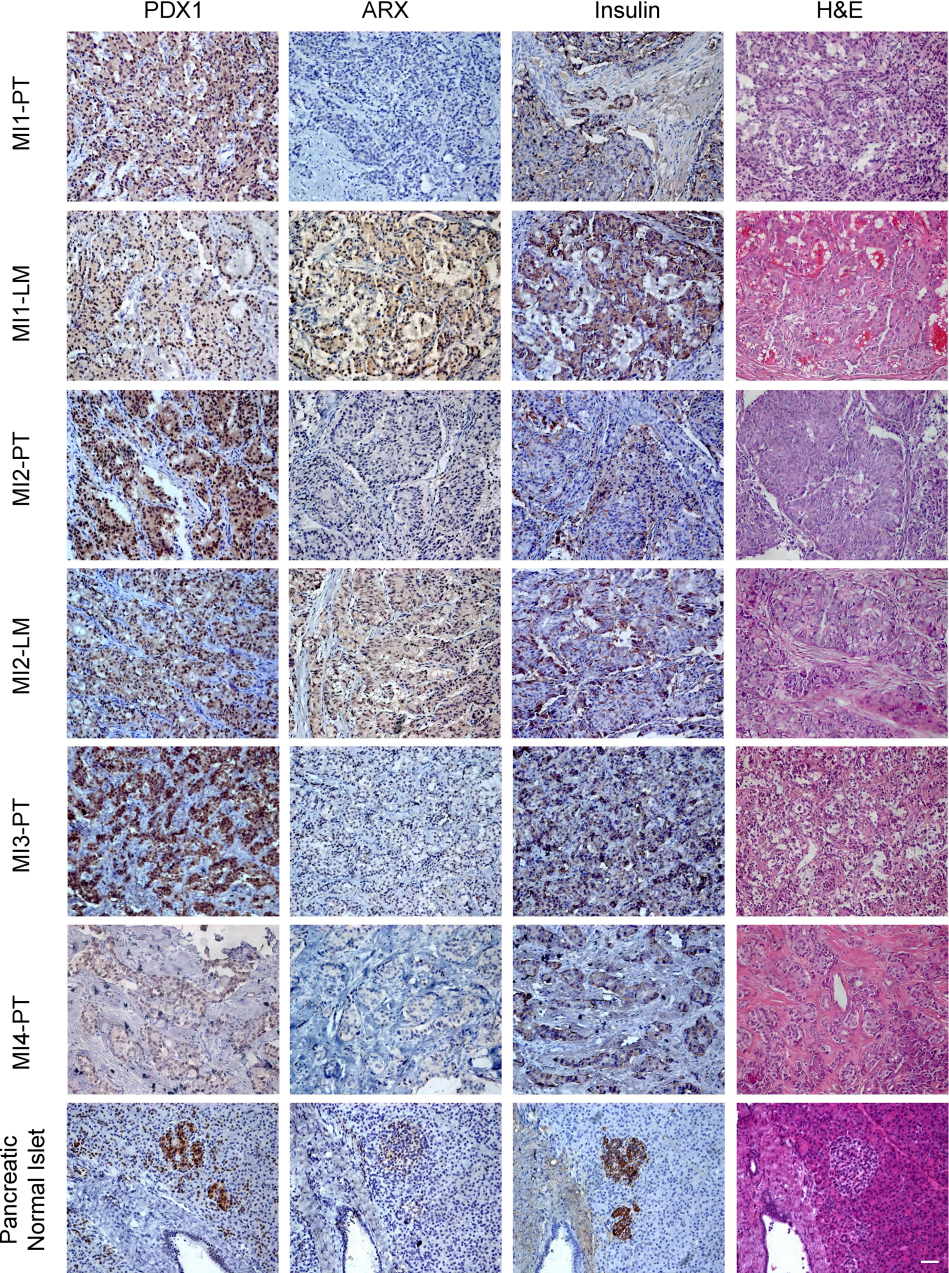
The H&E and immunohistochemical staining of ARX, PDX1, and Insulin of primary tumors and liver metastasis. PT, primary tumor. LM, liver metastasis. Scale bar, 100μm. H&E, haematoxylin and eosin staining.

Previous studies have shown that non-metastatic insulinomas exclusively expresses *PDX1*, whereas liver metastatic foci and metastatic insulinoma are more likely to express *ARX* (12, 16). In this study, all the primary tumors were PDX1+, ARX-, and insulin+, while liver metastasis foci of patients MI1 and MI2 both showed PDX1+, ARX+, and insulin+ (**Figure 3**).

**Figure 4:**
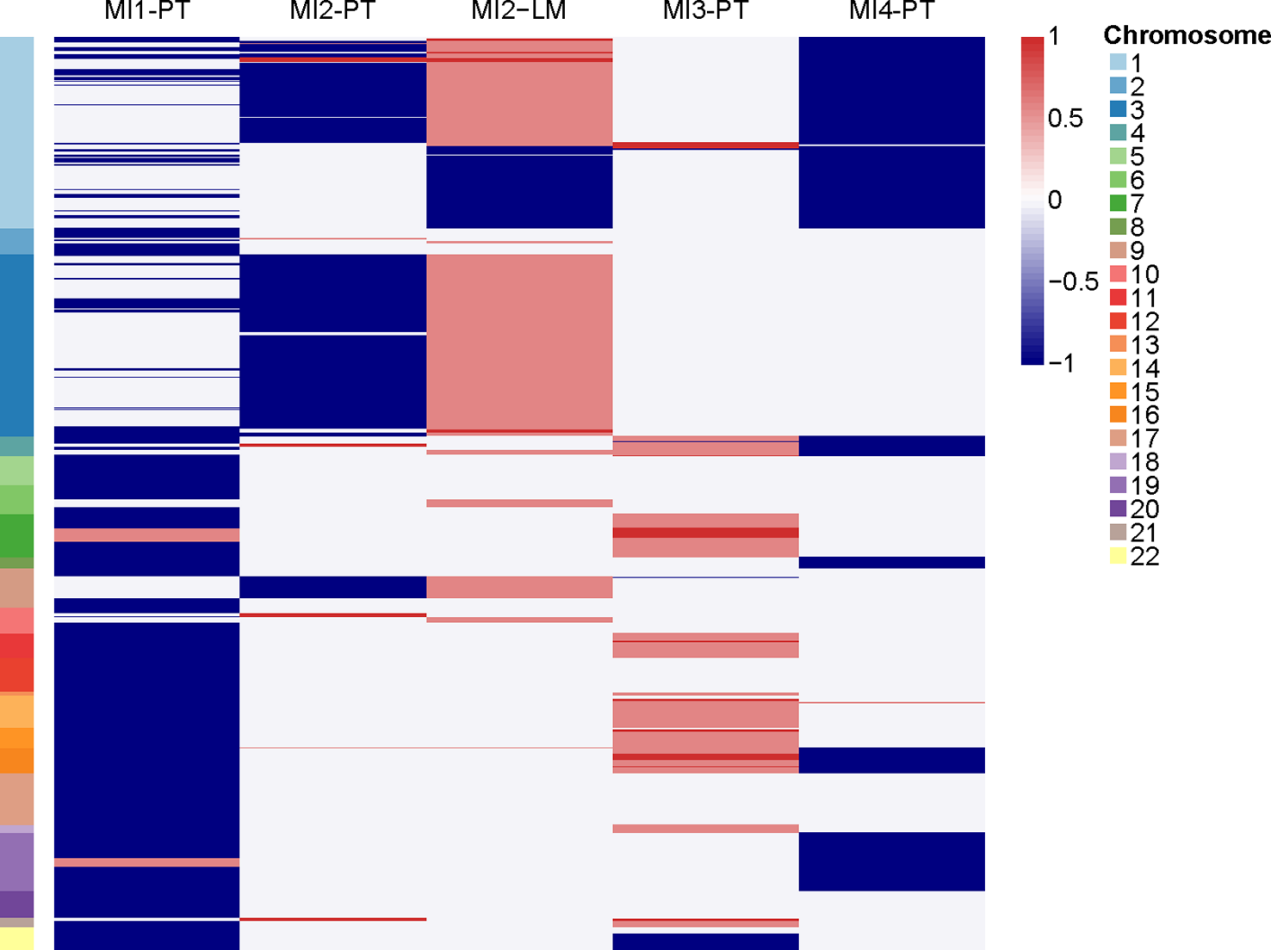
The copy number variations of primary tumors and liver metastasis. PT, primary tumor. LM, liver metastasis.

We performed WES and WGS on the primary tumors of the four patients and liver metastasis of MI2 to explore somatic gene mutations and CNVs. Genomic sequencing data showed that gene mutations previously detected in NF-PanNETs (such as *MEN1, DAXX*, and *ATRX*, etc) were not found in these four patients. However, MI4 harboured the *YY1* T372R mutation, which is a recurrently mutated gene in non-metastatic insulinomas. The non-silent mutations of these four patients were summarized in **Table 2**. However, it seems that there were no typical CNV pattern for these four patients. The typical CNV patterns of NF-PanNETs [chromosome loss of 1, 2, 3, 6, 8, 10, 11, 15, 16, 21, and 22, and chromosome gain of 4, 5, 7, 9, 12, 13, 14, 17, 18, 19, and 20 (18, 24)] were not found in these metastatic insulinomas (**Figure 4**).

**Table 2:** The non-silent gene mutations of four metastatic insulinomas patients.

Case	Number of mutations	Chromosome	Gene	Variant classification
MI1-PT	2	15	*TMEM62*	Missense_Mutation
		17	*GGT6*	Frame_Shift_Del
MI2-LM	3	2	*RETSAT*	Missense_Mutation
		3	*MUC20*	Missense_Mutation
		3	*MUC4*	Missense_Mutation (x3)
MI2-PT	6	7	*PAPOLB*	Missense_Mutation
		15	*GOLGA6L2*	Missense_Mutation
		16	*TBL3*	Missense_Mutation
		17	*WRAP53*	Missense_Mutation
		19	*OR7E24*	Frame_Shift_Del
		X	*MED12*	Missense_Mutation
MI3-PT	11	5	*SEPP1*	Missense_Mutation
		10	*MXI1*	Nonsense_Mutation
		11	*AHNAK*	Missense_Mutation
		11	*PRKRIR*	Missense_Mutation
		12	*BHLHE41*	Missense_Mutation
		16	*CDH8*	Missense_Mutation
		16	*CMTM2*	Splice_Site
		16	*EXOC3L1*	Splice_Site
		16	*WWP2*	Missense_Mutation
		22	*XRCC6*	Missense_Mutation
		X	*USP9X*	Nonsense_Mutation
MI4-PT	19	1	*SLC22A15*	Missense_Mutation
		2	*NHEJ1*	Missense_Mutation
		2	*FBXO11*	Missense_Mutation
		3	*MUC4*	Missense_Mutation
		3	*PIGX*	Missense_Mutation
		4	*SEC24B*	Missense_Mutation
		5	*GFPT2*	Splice_Site
		7	*HUS1*	Frame_Shift_Del
		8	*CPSF1*	Missense_Mutation
		9	*IARS*	Missense_Mutation
		10	*FBXL15*	Missense_Mutation
		11	*TMEM135*	De_novo_Start_OutOfFrame
		14	*YY1*	Missense_Mutation
		14	*KIF26A*	Missense_Mutation
		15	*DLL4*	Splice_Site
		19	*BRD4*	Missense_Mutation
		19	*ANKLE1*	Missense_Mutation
		22	*SBF1*	Missense_Mutation
		X	*CT45A5*	Missense_Mutation

## Conclusion

The natural history, circulating hormone levels, and IHC staining results indicate that primary tumors of these four metastatic insulinomas remained clinicopathological features of non-metastatic insulinomas, indicating it may largely derive from non-metastatic insulinomas other than NF-PanNETs. Nevertheless,

the liver metastasis showed ARX+ features resembling NF-PanNETs, indicating the accumulated ARX expression may play an important role in the progression of metastatic insulinomas.

## Discussion

Metastatic insulinoma is a rare type of PanNET that exhibits both malignant biological behaviours and excess hormone secretion patterns. Previous studies have shown that metastatic insulinoma has a longer duration of symptoms, higher insulin and C-peptide levels, and a larger tumor size. However, reliable indicators for the detection of metastatic insulinomas are still lacking. Some researchers have found that metastatic insulinomas may largely be derived from NF-PanNETs (8, 13), but it needs further exploration.

The preoperative elevated proinsulin levels and proinsulin/insulin molar ratio could predict the malignancy of insulinoma as well as ARX expression by IHC staining (23, 25). However, the hormone levels and IHC staining of primary tumors for these four metastatic insulinomas were similar to non-metastatic insulinomas, while liver metastasis showed signature of both α and β cell, indicating the complexity of metastatic insulinoma. The *ARX* and *PDX1* expression is necessary for α and β cell differentiation, respectively. PDX1 staining positive was commonly found in non-metastatic insulinomas, while ARX staining positive was preferred in a portion of NF-PanNETs. The primary tumors of these four patients all showed PDX1+ and ARX-, while the liver metastasis of patient MI1 and MI2 both showed PDX1+ and ARX+. It seems that the accumulated ARX expression was important for malignant signatures of metastatic insulinomas, but it needs further exploration.

To determine the molecular characteristics of metastatic insulinoma, we attempted to elucidate them through genomic sequencing. Only one patient harboured *YY1* T372R mutation, which is a recurrently mutated gene in non-metastatic insulinomas. We previously described somatic mutations of NF-PanNETs (*MEN1*, *DAXX*, *ATRX*, and mTOR pathway-related genes), but there was no such genetic mutation in these four metastatic insulinomas. Meanwhile, no typical signature of CNV patterns was found in these four metastatic insulinomas, which may be due to the limited cases. The typical CNV patterns of NF-PanNETs were also not shown in all NF-PanNET patients, the absence of the typical patterns in these four patients also make sense. Previous studies have indicated that chromosome 6q loss and 12q, 14q, and 17pq gains are strongly associated with metastatic insulinoma, whereas such CNV patterns was not found in our study.

For metastatic insulinoma, primary tumor should be resected to improve the life quality, and metastasis also need to be removed because the tumors could secrete excess insulin resulting in hypoglycemia symptoms. For instance, MI2 patient underwent resection of the pancreatic body and tail, but the metastasis could not be completely resected because of the extensive spread of the liver metastatic lesions. After surgery, the patient still suffered from hypoglycaemia, but the symptoms were immediately controlled when the interventional therapy for metastasis was performed.

At present, there is still a lot of debate regarding whether to enucleate insulinomas. Previous studies indicated that patients with liver metastasis before surgery generally choose pancreatectomy over enucleation. For insulinoma patients without metastasis before surgery, the proportion of metachronous metastasis after enucleation is approximately 2% (10). Currently, guidelines recommend active surgical treatment for insulinoma patients. Enucleation is only suitable for insulinoma with a diameter ≤ 2 cm and the distance > 2–3 mm from the main pancreatic duct, and rapid frozen pathological examination indicates that it is benign. However, predicting the occurrence of early metastasis before surgery remains difficult.

The limitation of this study is the small sample size. We only found four cases which met the criteria for metastatic insulinoma with clear Whipple symptoms, however, we basically collected all clinicopathological information and genomic sequencing data. More cases should be collected and analysed in the future. Moreover, the molecular mechanisms of metastatic insulinoma remain unclear because of its rarity, and transcriptomic and epigenetic changes need to be further elucidated.

## Data availability statement

According to national legislation/guidelines, specifically the Administrative Regulations of the People’s Republic of China on Human Genetic Resources (http://www.gov.cn/zhengce/content/2019-06/10/content_5398829.htm, http://english.www.gov.cn/policies/latest_releases/2019/06/10/content_281476708945462.htm), no additional raw data is available at this time. Data of this project can be accessed after an approval application to the China National Genebank (CNGB, https://db.cngb.org/cnsa/). Please refer to https://db.cngb.org/, or email: CNGBdb@cngb.org for detailed application guidance. The accession code CNP0000383 should be included in the application.

## Ethics statement

The studies involving human participants were reviewed and approved by Peking Union Medical College Hospital Ethical Review Committee. The patients/participants provided their written informed consent to participate in this study.

## Author contributions

JZ is responsible for article drafting and clinical data collection. RJ is responsible for immunohistochemical staining and sequencing data analysis. XHo was responsible for whole genome sequencing and whole exome sequencing of four tumors. HW is responsible for pathology examination. XHa and WW are responsible for coordinating the whole article, guiding and reviewing the article. XHa and WW are corresponding authors for this article. All authors contributed to the article and approved the submitted version.
